# Cardiac magnetic resonance T2 mapping and feature tracking in athlete’s heart and HCM

**DOI:** 10.1007/s00330-020-07289-4

**Published:** 2020-10-15

**Authors:** Mareike Gastl, Vera Lachmann, Aikaterini Christidi, Nico Janzarik, Verena Veulemans, Sebastian Haberkorn, Leonie Holzbach, Christoph Jacoby, Bernhard Schnackenburg, Susanne Berrisch-Rahmel, Tobias Zeus, Malte Kelm, Florian Bönner

**Affiliations:** 1 Department of Cardiology, Pulmonology and Vascular Medicine, Heinrich Heine University Düsseldorf, Moorenstraße 5, Düsseldorf, 40225 Germany; 2 Philips Healthcare, Hamburg, Germany; 3 KardioPro, Praxis für Innere Medizin, Kardiologie, Sport Medizin und Sportkardiologie, Düsseldorf, Germany; 4 CARID (Cardiovascular Research Institute Düsseldorf), Düsseldorf, Germany

**Keywords:** Multiparametric magnetic resonance imaging, Athletes, Hypertrophy, Left ventricular, Cardiomyopathy, hypertrophic

## Abstract

**Objectives:**

Distinguishing hypertrophic cardiomyopathy (HCM) from left ventricular hypertrophy (LVH) due to systematic training (athlete’s heart, AH) from morphologic assessment remains challenging. The purpose of this study was to examine the role of T2 mapping and deformation imaging obtained by cardiovascular magnetic resonance (CMR) to discriminate AH from HCM with (HOCM) or without outflow tract obstruction (HNCM).

**Methods:**

Thirty-three patients with HOCM, 9 with HNCM, 13 strength-trained athletes as well as individual age- and gender-matched controls received CMR. For T2 mapping, GRASE-derived multi-echo images were obtained and analyzed using dedicated software. Besides T2 mapping analyses, left ventricular (LV) dimensional and functional parameters were obtained including LV mass per body surface area (LVMi), interventricular septum thickness (IVS), and global longitudinal strain (GLS).

**Results:**

While LVMi was not significantly different, IVS was thickened in HOCM patients compared to athlete’s. Absolute values of GLS were significantly increased in patients with HOCM/HNCM compared to AH. Median T2 values were elevated compared to controls except in athlete’s heart. ROC analysis revealed T2 values (AUC 0.78) and GLS (AUC 0.91) as good parameters to discriminate AH from overall HNCM/HOCM.

**Conclusion:**

Discrimination of pathologic from non-pathologic LVH has implications for risk assessment of competitive sports in athletes. Multiparametric CMR with parametric T2 mapping and deformation imaging may add information to distinguish AH from LVH due to HCM.

**Key Points:**

*• Structural analyses using T2 mapping cardiovascular magnetic resonance imaging (CMR) may help to further distinguish myocardial diseases.*

• *To differentiate pathologic from non-pathologic left ventricular hypertrophy, CMR including T2 mapping was obtained in patients with hypertrophic obstructive/non-obstructive cardiomyopathy (HOCM/HNCM) as well as in strength-trained athletes.*

• * Elevated median T2 values in HOCM/HNCM compared with athlete’s may add information to distinguish athlete’s heart from pathologic left ventricular hypertrophy.*

## Introduction

Hypertrophic cardiomyopathy (HCM) has implications on risk assessment of competitive athletes due to cardiovascular complications including sudden cardiac death or arrhythmias [1]. As the incidence of HCM varies dependent on ethnicity and gender, cardiovascular diagnostic tests in professional athletes are warranted [2–4]. Left ventricular hypertrophy (LVH) hampers the diagnosis of HCM, as it is a common morphologic feature in high-performance athletes (athlete’s heart, AH). According to previous studies, AH is thought to represent a physiologic adaptation due to strength training with concentric hypertrophy (pressure overload) or due to endurance training with eccentric hypertrophy (volume overload) [5, 6]. Most sports yield a combination of both mechanisms introducing a mixture of concentric and eccentric hypertrophy respectively.

The American and European guidelines recommend the exclusion of athletes with HCM from competitive sports with the exception of low-intensity activity [1, 4, 7, 9]. This supports the need to distinguish different entities of LVH, especially HCM, from AH.

Besides clinical history, physical examination, and 12-lead electrocardiogram (ECG), transthoracic echocardiography (TTE) with a focus on morphologic and functional parameters is regularly performed in athlete assessment [7–9]. For certain phenotypes, there is still a grey zone between HCM and AH on the basis of pure morphological and global functional assessment [1, 10].

In cases of uncertainty, cardiovascular magnetic resonance (CMR) is recommended as an additional imaging approach due to its reproducibility as well as its ability for the characterization of myocardial structure, e.g., by using contrast agents [11]. Standard CMR exams with morphological and global functional analysis can be improved by the addition of myocardial deformation indices using feature tracking algorithm. Moreover, CMR covers the potential to characterize myocardial structure using myocardial magnetic relaxation properties (parametric mapping) without the need for contrast agents [12–14].

In this context, T2 values have been shown to increase diagnostic accuracy in myocarditis as they detect extracellular fluid imbalances [14]. For LVH, increased myocardial T2 values have previously been reported in patients with HCM, Fabry’s disease, or aortic stenosis [12, 15, 16]. As the role of T2 values in strength-trained AH is yet unknown, the purpose of this study was to examine the additional value of myocardial deformation analysis and parametric T2 mapping CMR to discriminate AH from a group of HCM with (HOCM) or without left ventricular outflow tract obstruction (HNCM).

## Materials and methods

The study was conducted in accordance to the Declaration of Helsinki and approved by the local ethics committee (application number 4307). Written informed consent was waived by the Institutional Review Board. Athletes gave their written informed consent.

### Participants

Thirty-three patients with HOCM (13 males, 60.5 ± 17.9 years), 9 patients with HNCM (7 males, 47.1 ± 8.3 years) and 13 strength-trained, healthy athletes with a minimum of 120 kg weightlift in the bench press (all males, 35.3 ± 12.2 years, mean weight lift: 155.4 ± 20.4 kg, mean years of training duration: 13.7 ± 8.6) received CMR. There was no history of cardiovascular diseases or a previous pathologic ECG in athletes. The inclusion criteria for HOCM and HNCM were based on the 2014 ESC Guidelines [17]. Strength-trained athletes were further excluded if the major pectoralis muscle was < 4 cm in diameter measured at the bifurcation of the pulmonary artery in CMR.

A group of age-, gender-, and comorbidity-matched volunteers to the different types of LVH, but no LVH itself, served as controls. Controls were ethnicity-matched as well. Volunteers were screened and included during routine cardiology consultations.

### CMR

CMR was performed on a 1.5-T MRI-system (Achieva, Philips) using a 32-channel phased array coil. After scout and reference scans, a gradient-spin echo (GRASE) sequence for T2 mapping was acquired as described previously [13]. Briefly, a stack of 15 images with increasing echo time (TE) (10-ms interecho-spacing) was acquired at end-diastole (3 slices, repetition time (TR): 1 cardiac cycle, flip angle 90°, spatial resolution 2 × 2 × 10 mm^3^, EPI factor 3, parallel imaging with an acceleration factor of 2).

Further functional and structural assessment was determined by cine steady-state free precession (SSFP) images in standard long-axis geometries (two-, three-, and four-chamber view) as well as in short-axis orientation with full ventricle coverage from basis to apex (TR/TE = 2.9/1.5 ms, FA = 60°, res = 8 × 1.5 × 1.5 mm^3^, 35 phases, breath-hold).

### Post processing

The SSFP short- and long-axis slices were analyzed according to left (LV) and right ventricular (RV) dimensional and functional parameters (Extended MK Work Space, Philips Medical Systems).

Myocardial feature tracking was performed offline using the Image-Arena software (Image-Arena VA Version 3.0 and 2D Cardiac Performance Analysis; TomTec Imaging Systems) [18]. Cine images were used for analyses of global longitudinal strain (GLS) from the long-axis stacks and peak early diastolic circumferential strain rate (SR_cc_) from the short-axis stack. Endocardial contours were applied followed by subsequent software-driven automatic tracking. Quality adjustment was performed and contours were amended manually if necessary.

GRASE images for local T2 value generation were post-processed using software based on the LabView environment (National Instruments) [13]. Endo- and epimyocardial contours were manually drawn as a region of interest (ROI) in the native images of the basal and midventricular short-axis slice. For every pixel within this ROI, the time constant of the signal intensity decay over all echoes was calculated by fitting a mono-exponential decay curve. Afterwards, median/mean T2 values and standard deviations (SD) were calculated for each segment of the AHA 16-segment model [19]. To avoid influence of high T2 values due to endocardial slow flow artefacts and epicardial fat, a limit of 110 ms was chosen.

### Statistical analysis

Statistical analysis was performed using SPSS 25.0 (SPSS Inc.). Unless otherwise stated, quantitative data are reported as mean ± SD. Normal distribution was tested using the Shapiro-Wilk test.

Continuous data were analyzed using analysis of variance (ANOVA) with the post hoc Bonferroni analysis to examine differences between the LVH groups for normally distributed data and the Kruskal-Wallis *H* test with post hoc Bonferroni correction for not normally distributed data. Data between the LVH groups and their respective controls were analyzed by 2-sided unpaired Student’s *t* tests for normally distributed data and the Mann-Whitney *U* test for not normally distributed data. Fisher’s exact test was used to examine significant differences between nominal classifications.

Receiver operating characteristics (ROC) were used to generate cutoff values to define sensitivity and specificity for the diagnosis of AH. *P* values below 0.05 were considered statistically significant.

## Results

### Baseline characteristics

The demographic and clinical characteristics of patients and controls are summarized in Table 1. Age of HNCM and AH cohorts was no different (*p* = 0.608) whereas patients with HOCM were significantly older according to post hoc testing (*p* < 0.01 to AH). Concomitant to an elevated left ventricular end-diastolic pressure, patients with HOCM and HNCM displayed a considerable amount of dyspnea in the New York Heart Association (NYHA) class III–IV (45% of patients with HOCM, and 11% in patients with HNCM, 0% in AH cohort).

**Table 1:** Demographic and clinical characteristics in all cohorts of LVH as well as in controls. *P* values indicate results from ANOVA analyses between groups of LVH

	LVH				Controls		
	HOCM	HNCM	AH	*p* value	HOCM controls	HNCM controls	AH controls
Demographics	*N* = 33	*N* = 9	*N* = 13		*N* = 33	*N* = 9	*N* = 13
Male, *n* (%)	13 (39)	7 (78)	13 (100)	< 0.001	13(39)	7 (78)	13 (100)
Age (years)	60.5 ± 17.9	47.1 ± 8.3	35.3 ± 12.2	< 0.001	66.8 ± 14.8	46.8 ± 8.0	32.3 ± 11.5
BMI (kg/m^2^)	28.0 ± 6.1	27.6 ± 4.8	29.6 ± 3.8	0.231	24.1 ± 3.0*	23.7 ± 3.0	22.1 ± 3.5^#^
Clinical							
Diabetes, *n* (%)	2 (6)	1 (11)	0 (0)	0.530	3 (9)	0 (0)	0 (0)
Manifest hypertension, *n* (%)	25 (78)	0 (0)	0 (0)	< 0.001	22 (67)	4 (44)	1 (8)
Hypercholesterolemia, *n* (%)	17 (53)	2 (22)	0 (0)	0.001	12 (36)	2 (22)	0 (0)
CKD III, *n* (%)	9 (28)	2 (22)	0 (0)	0.068	5 (15)	1 (11)	0 (0)
CAD, *n* (%)	7 (2)	1 (11)	0 (0)	0.200	6 (18)	1 (11)	0 (0)
PCI, *n* (%)	3 (9)	1 (11)	0 (0)	0.611	3 (9)	1 (11)	0 (0)
Class NYHA III–IV, *n* (%)	15 (45)	1 (11)	0 (0)	0.003	1 (3)*	0 (0)	0 (0)
MR > I°, *n* (%)	12 (36)	1 (11)	0 (0)	0.013	9 (27)	1 (11)	0 (0)

*ANOVA*, analysis of variance; *AH*, athlete’s heart; *BMI*, body mass index; *CAD*, coronary artery disease; *CKD III*, chronic kidney disease (glomerular filtration rate < 60 ml/min); *HNCM*, hypertrophic non-obstructive cardiomyopathy; *HOCM*, hypertrophic obstructive cardiomyopathy; *MR*, mitral regurgitation; *NYHA*, New York Heart Association classification; *PCI*, percutaneous coronary intervention. */^#^< 0.05/0.01 in comparison with the corresponding LVH group

### CMR characteristics

Table 2 displays baseline CMR parameters. There was no difference in heart rate (HR) and left ventricular mass indexed to body surface area (LVMi) among all entities of LVH. In comparison with their normal controls, LVMi was significantly elevated in all types of LVH (*p* < 0.05 all). Hypertrophy was predominantly localized in septal segments (segments 2, 3, 8, and 9) with a significantly thickened IVS in HOCM in the post hoc Bonferroni correction, but no difference between HNCM and AH.

**Table 2:** CMR characteristics in all cohorts of LVH as well as in controls. *p* values indicate the results from ANOVAs between groups of LVH

	LVH				Controls		
	HOCM	HNCM	AH	*p* value	HOCM controls	HNCM controls	AH controls
CMR	*N* = 33	*N* = 9	*N* = 13		*N* = 33	*N* = 9	*N* = 13
HR (bpm)	71.4 ± 13.3	63.3 ± 8.6	76.5 ± 13.4	0.060	77.1 ± 13.2	74.3 ± 4.0*	78.6 ± 15.5
LVEF (%)	72.3 ± 7.9	76.9 ± 6.7	58.4 ± 6.5	< 0.001	69.4 ± 7.2	67.6 ± 4.8^#^	64.7 ± 7.6*
IVS (mm)	21.7 ± 5.6	17.7 ± 4.4	15.1 ± 2.0	< 0.001	9.7 ± 2.4^#^	9.3 ± 2.2^#^	10.2 ± 1.7^#^
LVMi (g/m2^)^	91.1 ± 30.6	85.1 ± 25.9	73.6 ± 8.6	0.117	51.4 ± 15.3^#^	61.3 ± 11.1*	61.6 ± 10.4^#^
LVEDV (ml)	130.4 ± 44.2	137.7 ± 36.4	173.5 ± 28.2	0.003	118.9 ± 30.0	153.3 ± 39.2	161.3 ± 32.8
SV (ml)	93.4 ± 28.9	104.4 ± 22.4	91.5 ± 27.6	0.339	81.1 ± 17.5*	97.4 ± 16.1	99.0 ± 17.9
RVEF (%)	68.8 ± 6.3	64.4 ± 5.9	54.1 ± 6.3	< 0.001	65.0 ± 6.7*	60.2 ± 4.6	57.6 ± 7.1
RVEDV ( ml)	99.8 ± 30.1	120.8 ± 31.2	160.1 ± 30.2	< 0.001	104.3 ± 26.0	120.4 ± 20 .2	140.9 ± 28.1
GLS (%)	- 24.2 ± 4.8	- 28.0 ± 5.1	- 16.6 ± 3.2	< 0.001	- 27.1 ± 3.2*	- 26.5 ± 3.6	- 23.7 ± 4.0^#^
SR_cc_ (s^-1^)	1.7 ± 0.5	1.6 ± 0.4	1.8 ± 0.5	0.513	1.9 ± 0.4*	1.8 ± 0.3	2.0 ± 0.5
T2 values (ms)	62.9 ± 5.6	63.3 ± 5.6	57.1 ± 5.0	0.010	58.5 ± 4.5^#^	56.3 ± 4.0*	55.5 ± 3.5

*AH*, athlete’s heart; *bpm*, beats per minute; *CMR*, cardiovascular magnetic resonance; *GLS*, global longitudinal strain; *HNCM*, hypertrophic non-obstructive cardiomyopathy; *HOCM*, hypertrophic obstructive cardiomyopathy; *IVS*, interventricular septum; *LVEDV*, left ventricular end-diastolic volume; *LVEF*, left ventricular ejection fraction; *LVMi*, left ventricular mass per body surface area; *RVEDV*, right ventricular end-diastolic volume; *RVEF*, right ventricular ejection fraction; *SR*_*cc*_, peak early circumferential strain rate; *SV*, stroke volume. */^#^ *p* ≤ 0.05/0.01 in comparison with the corresponding LVH group

Late gadolinium enhancement (LGE) was detected in 18 patients (47%, 3 patients did not receive contrast agent; in one patient, images were non-diagnostic) of the HOCM/HNCM group. No myocardial scarring was seen in the 6 athletes who received contrast agent. LGE expression was not significantly different in Fisher’s exact test comparing HOCM/HNCM with AH (*p* = 0.067).

Left ventricular ejection fraction (LVEF) was significantly elevated in HOCM/HNCM compared with AH (AH vs. HOCM or HNCM: *p* < 0.001 both, Bonferroni correction) and between AH and their controls (*p* = 0.033). Accompanying the results for LVEF, absolute GLS was significantly elevated in patients with HOCM/HNCM compared with AH (*p* < 0.001 both, Bonferroni correction) (Table 2). GLS of HOCM and AH was significantly decreased compared with their controls (*p* = 0.025 for HOCM and *p* < 0.001 for AH). There was no difference in SR_cc_ between the different study cohorts. GLS and SR_cc_ of one HOCM subject had to be excluded due to poor image data, but no further data points of the other groups.

**Fig. 1:**
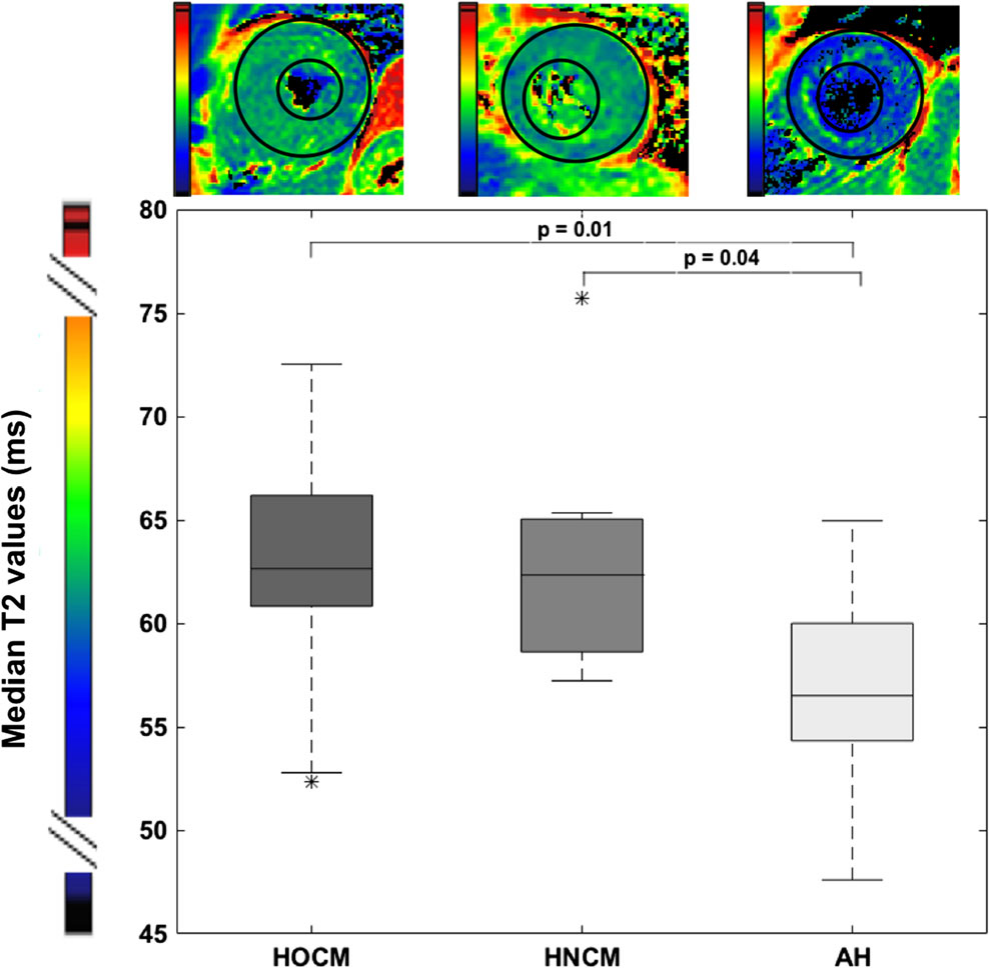
Global T2 values in all entities of LVH. Median T2 values of global myocardial analysis for patients with HOCM, HCNM, and AH. AH, athlete’s heart; HOCM, hypertrophic obstructive cardiomyopathy; HNCM, hypertrophic non-obstructive cardiomyopathy

As shown in Fig. 1, not only functional and morphological parameters were different between LVH-cohorts. Median global T2 values were significantly increased in patients with HOCM (*N* = 25 due to poor image quality of 8 subjects) and HNCM (*N* = 9) compared with AH (*N* = 12 due to poor image quality of one subject) (*p* = 0.014 for HOCM vs AH, *p* = 0.043 for HNCM vs. AH, both Bonferroni corrected).

Compared with their normal control groups, T2 values were significantly increased in LVH due to HOCM and HNCM (control HOCM: 58.5 ± 4.5 ms, *p* = 0.001; control HNCM: 56.3 ± 4.0 ms, *p* = 0.011). AH showed no difference from their respective controls (control AH: 55.5 ± 3.5 ms).

**Fig. 2:**
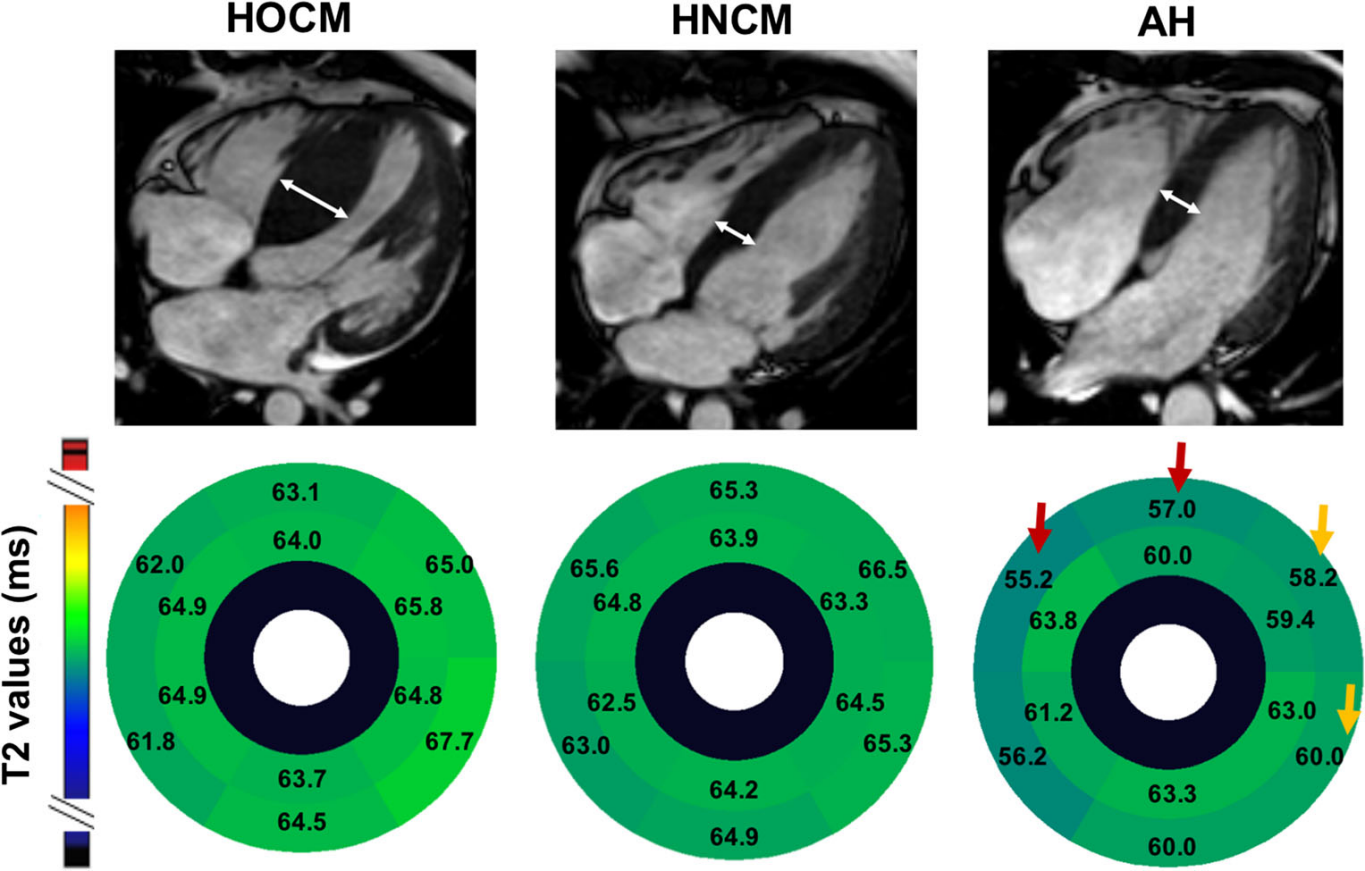
Segmental analysis of T2 values. Upper panel, end-diastolic 4-chamber cine view for the calculation of IVS (white double arrows). Lower panel, mean T2 value segmental analysis for patients with HOCM, HNCM, and AH. Red arrows indicate reduced T2 values compared with both entities of LVH, orange arrows to only HOCM. AH, athlete’s heart; HOCM, hypertrophic obstructive cardiomyopathy; HNCM, hypertrophic non-obstructive cardiomyopathy

In segmental analysis, the difference of T2 values was most indicated for the anterior and anteroseptal basal IVS (segments 1 and 2, *p* = 0.043 and 0.008 in overall ANOVA/Kruskal-Wallis *H* tests). This was most pronounced in segment 2 using the post hoc Bonferroni correction (HOCM vs. AH: 0.044, HNCM vs. AH: *p* = 0.010) (Fig. 2). There was also a difference in the inferolateral and anterolateral basal segments (segments 5 and 6) between HOCM and AH (*p* = 0.036 and *p* = 0.008).

To account for a dependency of GLS and T2 values to cofactors, Pearsons correlation was performed that showed a weak, but significant, correlation of T2 values to LVMi (*R* = 0.344, *p* = 0.019), HR (*R* = - 0.48, *p* = 0.001), and age (*R* = 0.52, *p* < 0.01). GLS showed a weak correlation to age (*R* = - 0.32, *p* = 0.017), but none to LVMi (*R* = - 0.025, *p* = 0.86). In addition, there was no impact of gender on T2 values (*p* = 0.056) or GLS (*p* = 0.328).

### Additional diagnostic testing

**Fig. 3:**
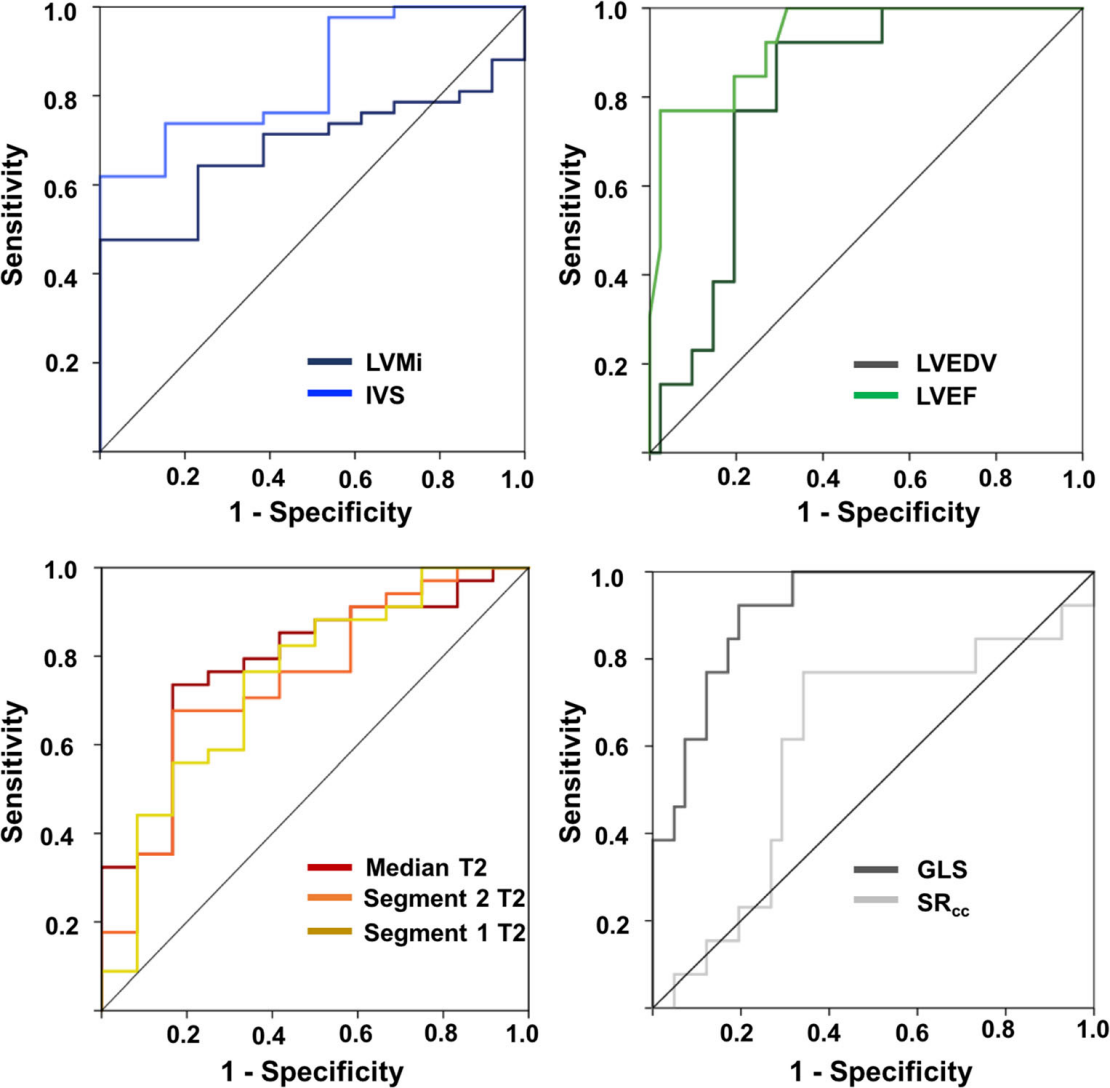
Receiver operating characteristics to differentiate AH from HOCM/HNCM. Besides IVS, LVEDV, and LVEF, AUC identified median T2 values (AUC = 0.78) and GLS (AUC = 0.91) as good parameters to differentiate between AH and pathologic LVH. AH, athlete’s heart; AUC, area under the curve; GLS, global longitudinal strain; IVS, interventricular septum; LVEDV, left ventricular end-diastolic volume; LVEF, left ventricular ejection fraction; LVH, left ventricular hypertrophy; LVMi, left ventricular mass per body surface area; *SR*_*cc*_, peak early circumferential strain rate

**Fig. 4:**
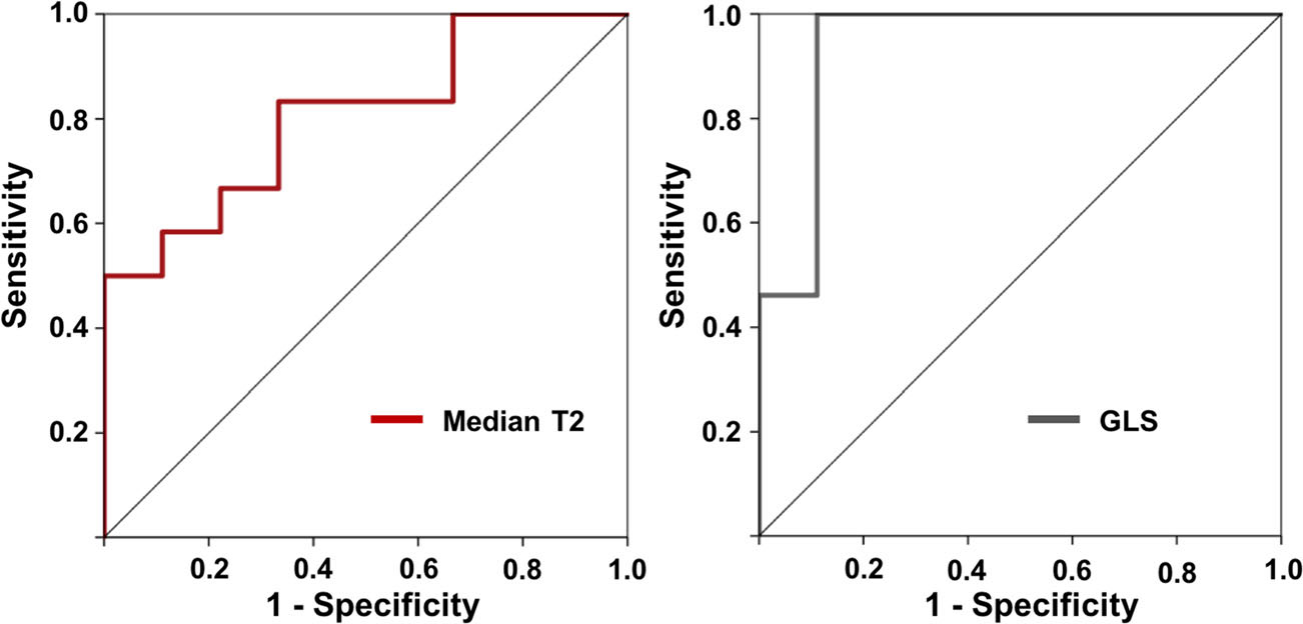
Receiver operating characteristics to differentiate AH from only HNCM. Area under the curve identified median T2 values (AUC = 0.81) and GLS (AUC = 0.94) as good parameters for the differentiation between HNCM and AH. AH, athlete’s heart; GLS, global longitudinal strain; HNCM, hypertrophic non-obstructive cardiomyopathy

The results of ROC analyses to differentiate pathologic LVH and AH are displayed in Fig. 3. Besides IVS (area under the curve (AUC) of 0.84), left ventricular end-diastolic volume (LVEDV) (AUC 0.81), and LVEF (AUC 0.93), ROC analyses identified T2 values and GLS as good parameters to differentiate AH from HOCM/HNCM (AUC with 0.78 and 0.91) (Fig. 3). A T2 cutoff value of > 59.9 discriminated AH with a sensitivity of 77% and a specificity of 75%. A GLS cutoff value was chosen at < 21.8% (sensitivity 92%, specificity 68%). ROC analysis of the sole comparison between HNCM and AH reproduced those results with an AUC for T2 values of 0.81 and for GLS of 0.94 (Fig. 4). A T2 cutoff value of > 61.4 ms (sensitivity 67%, specificity 83%) was most appropriate. Sensitivity and specificity increased for GLS to 89% and 100% using a cutoff value of > 24.3%. AUC of IVS was 0.62, of LVEDV 0.81, and of LVEF 0.97.

In further univariate regression analysis, LVEF (*p* < 0.01), IVS (*p* = 0.007), LVEDV (*p* = 0.007), GLS (*p* = 0.002), and T2 values (*p* = 0.007) were significant for the differentiation of AH from overall HCM. Multivariate analyses were not performed due to a potential overfit of the model as given by the low number of athletes and HNCM patients.

## Discussion

In the present study, absolute GLS and T2 values were elevated in HNCM and HOCM whereas AH exhibited normal T2 values. Based on these results, cutoff values for an additional differentiation between HCM from AH were identified. However, “classic” functional and dimensional parameters served as good parameters for a discrimination between the different LVH entities as well.

### Morphologic and functional CMR parameters

The American and European recommendations for pre-participation screening in athletes do not include TTE or CMR as standard methods. Those imaging modalities are consulted once suspicion of HOCM/HNCM arises due to far-reaching consequences in the exclusion of athletes from competitive sports [1, 9]. Although the definition of HCM is made in actual guidelines, diagnosis is hampered by genotype-phenotype correlations, such as a compatibility to any kind of wall thickness [17]. This results in a diagnostic grey zone of up to 18% in LVH of athletes [10, 20]. Previous CMR studies reported a diastolic wall-to-volume ratio of < 0.15 mm × m^2^ × ml^-1^ as the best discriminator between AH and other forms of LVH [7]. The study collective consisted of male athletes with a high level of dynamic sports training. In the present study, only 4 athletes had a ratio smaller than 0.15 mm × m^2^ × ml^-1^ as strength training leads to a more concentric LVH consequently increasing wall-to-volume ratio [11]. Compared with other studies, our participants were characterized by a higher percentage of thickened IVS, but a comparable LVMi [1, 21].

For feature tracking using CMR, there is no existing literature about the comparison of different LVH entities. As has been shown by TTE, diastolic parameters (E/A ratio, LV dyssynchrony) are good discriminators of HNCM and AH with the diastolic function being significantly more affected in HNCM [8, 22]. This finding could not be reproduced in our study, since diastolic function in AH and HOCM/HNCM (as measured by SR_cc_) was not significantly different. An explanation for this might be a heterogeneous course of HCM with a varying degree of diastolic dysfunction [23, 24]. In addition, a preclinical diastolic dysfunction could have been missed as CMR was only performed at rest, and TTE is assumed to be more sensitive to detect exercise-induced changes. Numbers in the HCM and AH groups could therefore be too low to detect subtle SR_cc_ changes. This is underlined by other measures of diastolic function in the HOCM/HNCM group that indicate diastolic function, e.g., the different NYHA class. Normal SR_cc_ values in athletes are in line with a previous CMR tagging study [25]. The reason for choosing peak early SR_cc_ to report diastolic function is given by its good reproducibility, especially in other entities of LVH, and its good correlation to measures of diastolic function from TTE [26–28].

Surprisingly, GLS was significantly decreased in AH compared to controls and the HCM cohort. Concerning GLS in AH, controversial literature exists. On the one hand, GLS values were decreased or equal in patients with HOCM/HCNM and AH, but within the same range as in the current study [29, 30], on the other hand, literature exists on reduced GLS in AH [31]. In general, this points towards heterogeneous findings in AH warranting further research. In the pathologic LVH group, GLS was decreased in patients with HOCM, but not different for HNCM. During the course of LVH in HOCM and HNCM, GLS and LVEF can still be preserved, which might underline a not decompensated course of the disease or a heterogeneous study cohort again [23].

### Myocardial magnetic relaxation analysis

Previous studies have highlighted the value of parametric mapping to distinguish different forms of cardiovascular diseases without the use of contrast agents. While T1 mapping is more likely to display diffuse myocardial fibrosis and local scarring, T2 mapping is subjected to display myocardial or interstitial water content [32, 33]. Data on T1 mapping in different forms of LVH exist and show a positive correlation of extracellular volume (ECV) to HCM and a negative correlation to AH [34]. In the present study, the focus was on T2 mapping and its implementation for diverse forms of LVH. As a primary result, T2 values showed feasibility to distinguish AH from the other entities of LVH. The elevated T2 values in HNCM/HOCM are in line with previous studies focusing on T2 mapping in LVH due to HCM and Fabry’s disease [12, 35]. In addition, T2 values were elevated in a group of patients with early dilated cardiomyopathy in comparison to a group with AH [36]. In the present study, the elevated T2 values were most prominent in the basal IVS, the preferred region of asymmetric LVH in HNCM/HOCM [11]. Although the present hypothesis was confirmed that there is a difference in T2 values between the HOCM/HNCM and AH group, one should keep in mind that there is still the potential of a statistical type II error, especially given the relatively low numbers in the HNCM and AH groups.

A possible explanation for the differences in T2 values might be found on a structural level. “Pathologic” LVH of HOCM/HNCM has been characterized by parallel addition of new sarcomeres, myofiber disarray, myocyte degeneration, and diffuse myocardial replacement fibrosis, e.g., due to small-vessel disease with relative ischemia [37–39]. Furthermore, elevated T2 values alongside an increased LVMi have already been used to monitor the effectiveness of enzyme treatment in LVH of Fabry’s disease [40]. In our cohorts of LVH, T2 values were only weakly correlated to LVMi prompting for additional influencing factors than cellular hypertrophy. That is why myocardial fluid imbalances (e.g. edema due to known relative ischemic reactions in HOCM and HNCM) can be suggested as further influencing parameters [41]. Myocardial fluid imbalances are known to elevate myocardial T2 values after myocardial infarction as well as in myocarditis [33, 42].

Age- and gender-related differences have already been reported as influencing factors on T2 values in healthy volunteers [13]. In our LVH cohort, a fair correlation between T2 values and age could be detected. Therefore, we included the older subjects of HOCM in our analyses, but still left a younger HNCM cohort. Although male gender was assigned lower T2 values in healthy volunteers, we could not detect an impact of gender on T2 values in our LVH cohort [13]. HR correlated negatively to T2 values as well. However, as the HOCM group had similar HR but higher T2 values and HR was not different between groups, no clear influence on the interpretation of the current results can be detected. Comorbidities such as hypertension and diabetes may influence T2 values as well, thereby hampering interpretation of the current results [13].

However, one should point out the overlap of T2 values between the different cohorts that may be triggered due to dynamic histopathological states of HOCM/HNCM with a varying degree of edema, cell death, fiber disarray, and myocardial scarring [43] as well as different ages or comorbidities. In addition, focal fibrosis has been described in athletes, e.g. at the insertion points of the right ventricle [44, 45]. Especially in the acute phases of myocardial alterations, this could influence and increase T2, thereby masking the differences of T2 values in the particular segments (segments 2, 3, 8, and 9). Although we additionally divided the AH group in athletes receiving contrast agents and excluding myocardial scarring and in a group with only T2 mapping with unknown scarring, there was still no difference in the particular segments in comparison with the HOCM/HNCM group. However, the focal fibrosis could still be too small for a substantial influence on a whole segment and further division of the single segments using mapping with a higher resolution should be pursued.

In addition, no difference could be seen in the LGE expression between the HOCM/HNCM and AH groups. This is likely due to the low number of athletes receiving contrast agent. LGE is still assumed as an essential component to differentiate the LVH groups, although focal fibrosis has been described at the insertion points of the right ventricle in athletes [45].

### Limitations

This is a single-center study and due to the small sample size, statistical analysis could have been hampered, especially for the small groups of HNCM and AH, which can be seen on the discrete data points of Fig. 4. Therefore, the results should be interpreted carefully. However, good reproducibility has been shown for global T2 and strain measurements as well as for segmental T2 mapping, thereby reducing a potential measurement bias introduced due to small numbers [13, 16, 46–48]. Before recruiting for the present study, we performed a power analysis based on our previous data [13, 15]. We thought to identify an effect in the difference of T2 values of HOCM/HNCM in comparison with T2 values of normal controls that we assumed in AH with a statistical power of 80% and a type I error of less than 5%. Under those conditions, the estimated sample size was 12. However, there is still the potential of a type II error, especially given the low numbers in the sub-groups of HNCM and AH. In addition, we summarized HNCM and HOCM in the diagnostic algorithm, as age only showed a fair linear relationship to T2 values. However, a multi-center study should strengthen the above results.

Our CMR protocol did not include systematic T1 mapping for completion of parametric mapping, since there is already convincing data of T1 values and AH [34, 49].

Athletes were recruited if they participated in physical strength training with a minimum weight lift of 120 kg in bench pressing. Despite explicitly neglecting the use of performance-enhancing substances such as steroids, we still could not control for substance abuse by blood sampling. It has been reported that those substances may have an impact on cardiovascular function and fibrosis [50, 51]. Due to the recruiting algorithm from fitness gyms, only male athletes could be included in this study meeting the inclusion criteria. As differences in gender exist for T2 mapping, inclusion criteria should be fitted on female athletes and a difference in T2 values compared with female HCM patients should further be observed [13]. In this context and by increasing numbers, a prospective validation of the present findings with respect to the cardiovascular outcome should be performed.

As we did not perform further testing such as catheterization, HNCM and other cardiovascular diseases could not be ruled out with absolute certainty for the athlete’s group. Based on the clinical history and cardiovascular symptoms, relevant cardiac diseases were excluded.

## Conclusions

Multiparametric CMR with parametric mapping identified preserved T2 values and reduced GLS of AH in comparison with elevated T2 values and preserved GLS in HCM. As the ability to differentiate between pathologic and non-pathologic LVH has implications for risk assessment and exclusion of competitive sports in athletes, this could be of importance as T2 mapping and GLS, besides other functional and dimensional LV parameters, can help to distinguish AH from HCM.

## Abbreviations

AH: Athlete’s heart
AUC: Area under the curve
ANOVA: Analysis of variance
CMR: Cardiovascular magnetic resonance
ECG: Electrocardiogram
ECV: Extracellular volume
GLS: Global longitudinal strain
GRASE: Gradient-spin echo
HCM: Hypertrophic cardiomyopathy
HNCM: Hypertrophic non-obstructive cardiomyopathy
HOCM: Hypertrophic obstructive cardiomyopathy
IVS: Interventricular septum
LVEDV: Left ventricular end-diastolic diameter volume
LVEF: Left ventricular ejection fraction
LVH: Left ventricular hypertrophy
LVMi: Left ventricular mass indexed per body surface area
NYHA: New York Heart Association
ROC: Receiver operating characteristics
ROI: Region of interest
RVEDV: Right ventricular end-diastolic diameter volume
RVEF: Right ventricular ejection fraction
SD: Standard deviation
SR_cc_: Peak early diastolic circumferential strain rate
SSFP: Steady state free precession
SV: Stroke volume
TE: Echo time
TR: Repetition time

## References

[1] Maron BJ (2005). Distinguishing hypertrophic cardiomyopathy from athlete’s heart: a clinical problem of increasing magnitude and significance. Heart.

[2] Maron BJ (2003). Sudden death in young athletes. N Engl J Med.

[3] Maron BJ, Shirani J, Poliac LC, Mathenge R, Roberts WC, Mueller FO (1996) Sudden death in young competitive athletes. JAMA 276:199–204. 10.1001/jama.1996.03540030033028 8667563

[4] Pelliccia A, Zipes DP, Maron BJ (2008). Bethesda Conference #36 and the European Society of Cardiology Consensus Recommendations Revisited: A Comparison of U.S. and European Criteria for Eligibility and Disqualification of Competitive Athletes With Cardiovascular Abnormalities. J Am Coll Cardiol.

[5] Cardim N, Galderisi M, Edvardsen T (2015). Role of multimodality cardiac imaging in the management of patients with hypertrophic cardiomyopathy: an expert consensus of the European Association of Cardiovascular Imaging Endorsed by the Saudi Heart Association. Eur Heart J Cardiovasc Imaging.

[6] Morganroth J, Maron BJ, Henry WL, Epstein SE (1975). Comparative left ventricular dimensions in trained athletes. Ann Intern Med.

[7] Petersen SE, Selvanayagam JB, Francis JM (2005). Differentiation of athlete’s heart from pathological forms of cardiac hypertrophy by means of geometric indices derived from cardiovascular magnetic resonance. J Cardiovasc Magn Reson.

[8] Ternacle J, Bremont C, D’Humieres T (2017). Left ventricular dyssynchrony and 2D and 3D global longitudinal strain for differentiating physiological and pathological left ventricular hypertrophy. Arch Cardiovasc Dis.

[9] Pelliccia A, Fagard R, Bjørnstad HH (2005). Recommendations for competitive sports participation in athletes with cardiovascular disease: a consensus document from the Study Group of Sports Cardiology of the Working Group of Cardiac Rehabilitation and Exercise Physiology and the Working Group of Myocardial and Pericardial Diseases of the European Society of Cardiology. Eur Heart J.

[10] Chandra N, Bastiaenen R, Papadakis M, Sharma S (2013). Sudden cardiac death in young athletes. J Am Coll Cardiol.

[11] Galderisi M, Cardim N, D’Andrea A (2015). The multi-modality cardiac imaging approach to the athlete’s heart: an expert consensus of the European Association of Cardiovascular Imaging. Eur Heart J Cardiovasc Imaging.

[12] Imbriaco M, Spinelli L, Cuocolo A (2007). MRI characterization of myocardial tissue in patients with Fabry’s disease. AJR Am J Roentgenol.

[13] Bönner F, Janzarik N, Jacoby C (2015). Myocardial T2 mapping reveals age- and sex-related differences in volunteers. J Cardiovasc Magn Reson.

[14] Spieker M, Haberkorn S, Gastl M (2017). Abnormal T2 mapping cardiovascular magnetic resonance correlates with adverse clinical outcome in patients with suspected acute myocarditis. J Cardiovasc Magn Reson.

[15] Bönner F, NeizelM, Gruenig S, Jacoby C, Kelm M, Sievers B (2013) T2 mapping in different cardiomyopathies: first clinical experience. J Cardiovasc Magn Reson 15:P53. 10.1186/1532-429X-15-S1-P53

[16] Gastl M, Behm P, Haberkorn S (2018). Role of T2 mapping in left ventricular reverse remodeling after TAVR. Int J Cardiol.

[17] Elliott PM, Anastasakis A, Authors/Task Force members (2014). 2014 ESC Guidelines on diagnosis and management of hypertrophic cardiomyopathy. Eur Heart J.

[18] Hor KN, Gottliebson WM, Carson C (2010). Comparison of magnetic resonance feature tracking for strain calculation with harmonic phase imaging analysis. JACC Cardiovasc Imaging.

[19] Cerqueira MD, Weissman NJ, Dilsizian V (2002). Standardized myocardial segmentation and nomenclature for tomographic imaging of the heart. A statement for healthcare professionals from the Cardiac Imaging Committee of the Council on Clinical Cardiology of the American Heart Association. Circulation.

[20] Watkins H, McKenna WJ, Thierfelder L (1995). Mutations in the genes for cardiac troponin T and α-tropomyosin in hypertrophic cardiomyopathy. N Engl J Med.

[21] Pluim BM, Zwinderman AH, van der Laarse A, van der Wall EE (2000). The athlete’s heart. A meta-analysis of cardiac structure and function. Circulation.

[22] Kreso A, Barakovic F, Medjedovic S, Halilbasic A, Klepic M (2015) Echocardiography Differences between athlete’s heart hearth and hypertrophic cardiomyopathy. Acta Inform Med 23:276. 10.5455/aim.2015.23.276-279 10.5455/aim.2015.23.276-279 PMC4639332 26635434

[23] Melacini P, Basso C, Angelini A (2010). Clinicopathological profiles of progressive heart failure in hypertrophic cardiomyopathy. Eur Heart J.

[24] Olivotto I, Cecchi F, Poggesi C, Yacoub MH (2012). Patterns of disease progression in hypertrophic cardiomyopathy. Circ Heart Fail.

[25] Swoboda PP, Erhayiem B, Mcdiarmid AK (2016). Relationship between cardiac deformation parameters measured by cardiovascular magnetic resonance and aerobic fitness in endurance athletes. J Cardiovasc Magn Reson.

[26] Kowallick JT, Morton G, Lamata P (2016). Inter-study reproducibility of left ventricular torsion and torsion rate quantification using MR myocardial feature tracking. J Magn Reson Imaging.

[27] Singh A, Steadman CD, Khan JN (2015). Intertechnique agreement and interstudy reproducibility of strain and diastolic strain rate at 1.5 and 3 tesla: a comparison of feature-tracking and tagging in patients with aortic stenosis. J Magn Reson Imaging.

[28] Chen S, Yuan J, Qiao S, Duan F, Zhang J, Wang H (2014) Evaluation of left ventricular diastolic function by global strain rate imaging in patients with obstructive hypertrophic cardiomyopathy: a simultaneous speckle tracking echocardiography and cardiac catheterization study. Echocardiography 31:615–622. 10.1111/echo.12424 10.1111/echo.12424 24219240

[29] Schnell F, Matelot D, Daudin M (2017). Mechanical dispersion by strain echocardiography: a novel tool to diagnose hypertrophic cardiomyopathy in athletes. J Am Soc Echocardiogr.

[30] Butz T, van Buuren F, Mellwig KP (2011). Two-dimensional strain analysis of the global and regional myocardial function for the differentiation of pathologic and physiologic left ventricular hypertrophy: a study in athletes and in patients with hypertrophic cardiomyopathy. Int J Cardiovasc Imaging.

[31] D’Andrea A, Bossone E, Radmilovic J (2015). The role of new echocardiographic techniques in athlete’s heart. F1000Res.

[32] Messroghli DR, Nordmeyer S, Dietrich T (2011). Assessment of diffuse myocardial fibrosis in rats using small-animal look-locker inversion recovery T1 mapping. Circ Cardiovasc Imaging.

[33] Messroghli DR, Moon JC, Ferreira VM (2017). Clinical recommendations for cardiovascular magnetic resonance mapping of T1, T2, T2* and extracellular volume: a consensus statement by the Society for Cardiovascular Magnetic Resonance (SCMR) endorsed by the European Association for Cardiovascular Imaging (EACVI). J Cardiovasc Magn Reson.

[34] Swoboda PP, McDiarmid AK, Erhayiem B (2016). Assessing myocardial extracellular volume by T1 mapping to distinguish hypertrophic cardiomyopathy from athlete’s heart. J Am Coll Cardiol.

[35] Kolman L, Stirrat J, Rajchl M (2014). Myocardial T2 signal enhancement in hypertrophic cardiomyopathy: prevalence, clinical profile and pathologic correlation. J Cardiovasc Magn Reson.

[36] Mordi I, Carrick D, Bezerra H, Tzemos N (2016). *T*_1_ and *T*_2_ mapping for early diagnosis of dilated non-ischaemic cardiomyopathy in middle-aged patients and differentiation from normal physiological adaptation. Eur Heart J Cardiovasc Imaging.

[37] Lorell BH, Carabello BA (2000). Left ventricular hypertrophy: pathogenesis, detection, and prognosis. Circulation.

[38] Maron BJ (2002). Hypertrophic cardiomyopathy: a systematic review. JAMA.

[39] Cecchi F, Olivotto I, Gistri R, Lorenzoni R, Chiriatti G, Camici PG (2003) Coronary microvascular dysfunction and prognosis in hypertrophic cardiomyopathy. N Engl J Med 349:1027–1035. 10.1056/NEJMoa025050 10.1056/NEJMoa025050 12968086

[40] Messalli G, Imbriaco M, Avitabile G (2012). Role of cardiac MRI in evaluating patients with Anderson-Fabry disease: assessing cardiac effects of long-term enzyme replacement therapy. Radiol Med.

[41] Maron MS, Olivotto I, Maron BJ (2009). The case for myocardial ischemia in hypertrophic cardiomyopathy. J Am Coll Cardiol.

[42] Bönner F, Spieker M, Haberkorn S (2016). Myocardial T2 mapping increases noninvasive diagnostic accuracy for biopsy-proven myocarditis. JACC Cardiovasc Imaging.

[43] Knaapen P, van Dockum WG, Götte MJW (2006). Regional heterogeneity of resting perfusion in hypertrophic cardiomyopathy is related to delayed contrast enhancement but not to systolic function: a PET and MRI study. J Nucl Cardiol.

[44] Tahir E, Starekova J, Muellerleile K (2018). Myocardial fibrosis in competitive triathletes detected by contrast-enhanced CMR correlates with exercise-induced hypertension and competition history. JACC Cardiovasc Imaging.

[45] Banks L, Altaha MA, Yan AT et al (2020) Left ventricular fibrosis in middle-age athletes and physically active adults. Med Sci Sports Exerc. 10.1249/MSS.0000000000002411 10.1249/MSS.0000000000002411 32472930

[46] Gastl M, Gotschy A, Polacin M et al (2019) Determinants of myocardial function characterized by CMR-derived strain parameters in left ventricular non-compaction cardiomyopathy. Sci Rep 9. 10.1038/S41598-019-52161-1 10.1038/s41598-019-52161-1 PMC6828801 31685845

[47] von Knobelsdorff-Brenkenhoff F, Prothmann M, Dieringer MA (2013). Myocardial T1 and T2 mapping at 3 T: reference values, influencing factors and implications. J Cardiovasc Magn Reson.

[48] Andre F, Steen H, Matheis P (2015). Age- and gender-related normal left ventricular deformation assessed by cardiovascular magnetic resonance feature tracking. J Cardiovasc Magn Reson.

[49] Görmeli CA, Görmeli G, Yağmur J (2016). Assessment of myocardial changes in athletes with native T1 mapping and cardiac functional evaluation using 3 T MRI. Int J Cardiovasc Imaging.

[50] Angell PJ, Chester N, Green DJ (2012). Anabolic Steroid use and longitudinal, radial, and circumferential cardiac motion. Med Sci Sports Exerc.

[51] Payne JR, Kotwinski PJ, Montgomery HE (2004). Cardiac effects of anabolic steroids. Heart.

